# Accessibility to manage the obstructive sleep apnea within the Brazilian Unified Health System^☆^

**DOI:** 10.1016/j.bjorl.2023.101338

**Published:** 2023-10-12

**Authors:** Douglas Inomata Cardoso da Silva, Camila de Castro Corrêa, Jefferson Luis de Barros, Antonio Carlos Marão, Silke Anna Theresa Weber

**Affiliations:** a Universidade Estadual de São Paulo (UNESP), Faculdade de Medicina de Botucatu, Botucatu, SP, Brazil; b Centro Universitário Planalto do Distrito Federal (UNIPLAN), Brasília, DF, Brazil

**Keywords:** Obstructive sleep apnea syndrome, Polysomnography, Diagnosis, Treatment, Healthcare system

## Abstract

**Objective:**

To measure the average time for the diagnosis and for the therapeutic prescription of Continuous Positive Airway Pressure (CPAP) at a hospital in Botucatu Medical School - State University São Paulo, UNESP.

**Method:**

A retrospective observational study was carried out by collecting data from the electronic medical records of patients over 18-years of age, who had a diagnostic polysomnography testing scheduled between January and December 2017.

**Result:**

Of the 347 patients eligible for the study, 94 (27.1%) missed follow-up and 103 (29.7%) had a referral for CPAP use. Until February 2021, only 37 (35.9%) of these patients had already acquired and were using the device, the remaining 66 (64.1%) were waiting or gave up the therapy. The mean value of the waiting time interval between the referral of the diagnostic test and its performance was equivalent to 197 days (6.5 months). The mean time between diagnostic polysomnography and CPAP prescription was 440-days (14.5-months), with a total mean time of 624 days (21-months).

**Conclusion:**

As in other services, the diagnostic-therapeutic flow proved to be highly inefficient, with a long waiting period, difficult access to treatment and a high dropout rate. These findings highlight the need to establish new patient-centered strategies with measures that speed up the flow and facilitate access to CPAP, in order to reduce the morbidity and mortality associated with this condition.

**Level of evidence:**

Level 3 - Non-randomized controlled cohort/follow-up study Recommendation B.

## Highlights

• A follow-up of the journey of the individual with OSA was carried out.
• 64.1% of the participants had still not used CPAP or had given up on therapy.
• Epidemiological and polysomnographic factors did not interfere with the use of CPAP.

## Introduction

The Obstructive Sleep Apnea (OSA) is the most prevalent sleep-related breathing disorder, affecting about 54% from the population at large.^1^ In Brazil, a cohort study was conducted in the city of São Paulo, finding 32.9% of general OSA prevalence, with 40.6% among males and 26.1% among females.^2^

OSA is a chronic condition featuring complete or partial superior airways obstruction, causing recurring apnea or hypopnea episodes during sleep. The prevalent OSA signs and symptoms are snoring, perceived apneas and Excessive Daytime Sleepiness (EDS), a major alleged cause of morbimortality. Scientific evidence points to the OSA as a contributor to the development of systemic arterial hypertension, cardiovascular and metabolic diseases among untreated chronic patients.^3^ It is associated with cognitive dysfunction (difficulty in learning and memory), mood disorders (depression and anxiety), poor performance at work, greater risks of car accidents and reduction in the quality of life.^4^

Clinical suspicion is given by anamnesis and patient’s physical examination, and diagnosis is performed by means of the overnight polysomnography (PSG), considered the gold standard method.^5^, ^6^ The gold standard treatment for moderate to severe OSA is the use of the Continuous Positive Airway Pressure (CPAP), a machine that generates and directs positive airway pressure by wearing a nasal or oronasal mask tightly fixed to the face, preventing its collapse. Prospective studies showed untreated OSA patients with higher mortality rate when compared to the general population and to patients who make use of a CPAP device.^3^, ^7^, ^8^

However, one of the major difficulties is the access to the diagnosis. In Brazil, there are scarce specialized centers at Unified Health System (UHS), which provide overnight PSG, thus not supplying the real demand for such services. Treatment is another barrier, despite the recognized efficiency of the Positive Airway Pressure (PAP) therapy. The CPAP is a costly equipment, not available to all of those whose use is recommended. A way to measure the efficacy to diagnosis and treatment would be to assess the mean waiting time for the diagnosis, between the first appointment with a Sleep Medicine specialist and the PSG diagnosis, and the waiting time interval for the patient’s access to the therapeutic prescription of the CPAP.

Nevertheless, national and international data related to those waiting time intervals have been scarce in the scientific literature. Knowledge on such mean time intervals is extremely relevant, once it enables to assess the patients’ flow speed, fundamental for the healthcare service to identify probable failures which hinder its work, in addition to the formulation of public policies in order to expand investments and services related to the OSA diagnosis and treatment, which would facilitate and speed diagnosis, reducing the waiting time to begin treatment, and consequently, this condition morbimortality rate. This study aims to measure the mean waiting time for the OSA diagnosis and therapeutic CPAP prescription at a reference public hospital in Botucatu Medical School - State University São Paulo, UNESP.

## Methods

The research project was approved by the local Research Ethics Board (Ethical Appreciation Certificate Research Ethics Board (Ethical Appreciation Certificate: 6451020.5.0000.5411, and opinion number: 4,267,775). It is a study with adult participants who scheduled and/or underwent the diagnostic PSG at the Hospital Sleep Laboratory of the aforementioned institution, reference in the interior of São Paulo State, Brazil, between January and December 2017. Individuals under 18-years of age, suffering from neuromuscular diseases, and simultaneous CPAP titration and/or oxygen during the diagnostic PSG testing were excluded.

The sociodemographic data, such as gender, age, and Body Mass Index (BMI), were collected, apart from the clinical profile, the presence of comorbidities, such as obesity, systemic arterial hypertension, dyslipidemia, diabetes mellitus, thyroidopathy and chronic obstructive pulmonary disease.

Retrospective data collection and analysis were actively conducted by means of review of patients’ records from the electronic medical record system, accessed between October 2020 and February 2021.

In the first triage, it was considered the participants who had an initial appointment at the sleep-related outpatient clinic, named as the phase 1 of the selection. Phase 2 contemplated the analysis of the participants who scheduled their PSG testing. Phase 3 addressed the PSG testing result itself. Finally, phase 4 contemplated the search for treatment.

The waiting time in terms of days was measured for the OSA diagnosis and the therapeutic prescription of the CPAP, as follows:

T0: time interval from the first appointment at the outpatient clinic of sleep-related disorders to the overnight PSG testing;

T1: time interval between the PSG testing and the first medical reappointment;

T2: time interval between the first medical reappointment and the CPAP titration;

T3: time interval between the titration and the CPAP prescription;

T4: time interval between the CPAP prescription and the beginning of its use.

The triage questionnaires were also assessed, previously responded to the diagnostic PSG, such as the Epworth Sleepiness Scale, the Berlin Questionnaire and the Pittsburgh Quality Sleep Index. The Epworth Sleepiness Scale evaluates the sleep probabilities in eight situations involving daytime activities. Scoring ranges from 0 to 24, with scores over 10 being suggestive to EDS.

The Pittsburg Sleep Quality Index assesses the sleep quality and the presence of probable sleep disturbances over one-month time. It considers seven components: subjective sleep quality, sleep latency, sleep duration, sleep efficiency, sleep disturbance, use of sleeping medication and daytime dysfunction. For each component, scores range from 0 to 3, global score from 0 to 21. Scores above 5 points to subjects’ poor sleep quality.

The Berlin Questionnaire was used to investigate the risk of developing OSA. It comprises questions divided into 3 categories: category 1 (snoring and perceived apnea), category 2 (EDS and fatigue), and category 3 (presence or not of obesity, by means of the BMI and SAH history). When individuals match the criteria of two or three of the assessed categories, they are considered at high risk of OSA.

Regarding data from the diagnostic PSG testing, the analysis of the Apnea-Hypopnea Index (AHI), Obstructive Apnea Index (OAI), Oxygen Desaturation Index (ODI), and partial pressure < 85% was performed. All the PSGs were scored and reported by the same team, based on the guidelines of the American Academy of Sleep Medicine (AASM).^9^ Considering the CPAP titration, the ideal pressure was verified, with the elimination of 90% of the obstructive events. Finally, among all participants with the machine prescription, it was assessed how many of them could get the device until the end of this study.

The analyzed variables in the study were described according to the absolute and relative frequencies concerning gender, comorbidities, presence of OSA and risk of OSA by the Berlin questionnaire. Mean age and age standard deviation for BMI, age, Epworth Scale, Pittsburgh Sleep Quality Index, AHI, OAI and ODI were used. In order to investigate clinical, epidemiological and polysomnographic differences among the subjects who started and did not start the use of the CPAP, both groups were compared by means of the Mann-Whitney statistical test (non-normal distribution), considering *p* < 0.05.

## Results

**Figure 1:**
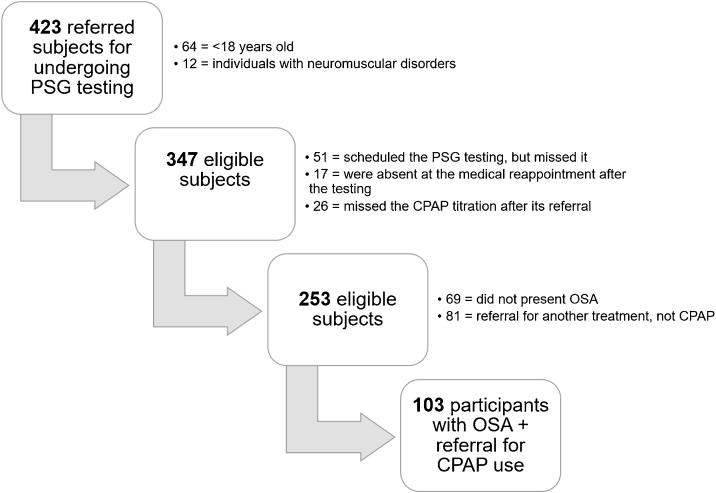
Flowchart of the patient’s trajectory who initially reported sleep complaints from a service connected with the Unified Health System in the interior of São Paulo State.

From 423 participants who scheduled the PSG, 76 were excluded. Phase 2 of the selection initially comprised 347 participants with scheduled PSG, but the exclusion of 188 participants was observed fundamentally for missing the scheduled date for undergoing the PSG testing at the sleep laboratory (14.7%). Phase 3 included 253 participants, among them, 19.8% were not diagnosed with OSA, and 23.3% were referred to another treatment unlike CPAP (Fig. 1).

Therefore, the case study after phase 4 entailed 103 participants with OSA and referral to CPAP titration, who underwent all the phases, that is to say, from the recommendation for undergoing the overnight PSG testing up to the device prescription. Regarding their age, mean age was 55.6 ± 11.8 years, minimum of 23 and maximum of 91-years old (Table 1).

**Table 1:** Clinical-epidemiological profile of the participants included in phase 4.

Profile	n = 103
Gender	
Male	54 (52.4%)
Female	49 (47.6%)
BMI (kg/m^2^)	34.6 (±7.2)
Clinical comorbidities	
Obesity	80 (77.7%)
Systemic arterial hypertension	73 (70.9%)
Dyslipidemia	62 (60.2%)
Diabetes mellitus	45 (43.7%)
Thyropathy	30 (29.1%)
Chronic obstructive pulmonary disease	15 (14.6%)

N, Number of participants; BMI, Body Mass Index.

Concerning the questionnaires, 86.4% of the participants scored high risk of OSA according to the Berlin questionnaire; 80% presented EDS according to the Epworth Scale, and 71.8% scored poor sleep quality by applying the Pittsburgh Sleep Quality Index. The mean AHI of the participants was 41.4 ± 26.3 events per hour, with OSA rated as severe (AHI ≥ 30) for 61.2% of them (Table 2).

**Table 2:** Characterization of the questionnaires, polysomnography testing and CPAP titration of the participants referred to this treatment.

Characteristics	n = 103
*Sleep questionnaires*	
Berlin questionnaire	No risk 0.97% (n = 1)
	Low risk 12.62% (n = 13)
	High risk 86.4 (n = 89)
ESS	12.82 ± 5.02
PSQI	15.97 ± 7.65
*Diagnostic with polysomnography*	
AHI (events/hour)	41.4 (±26.3)
OAI (events/hour)	17 (±20)
ODI	39.8 (±26.3)
Time (min) < Sat O_2_ 85%	54 (±24.4)
CPAP titration	
Ideal pressure (mmHg)	12.2 (±2.7)

ESS, Epworth Sleep Scale; PSQI, Pittsburgh Sleep Quality Index; AHI, Apnea-Hypopnea Index; OAI, Obstructive Apnea Index; ODI, Oxygen desaturation index.

The mean value of the waiting time interval between the testing referral and undergoing it (T0) was 197.3 ± 126.6 days (6.5-months). The mean time between the diagnostic PSG testing and the CPAP prescription (T1 + T2 + T3) was 440 ± 253.7 days (14.5-months), with total mean time of 623.9 ± 292.2 days (21-months). Until February 2021, only 35.9% (n = 37) of those participants had already obtained and were using the CPAP device; the remaining 64.1% (n = 66) of the subjects were still waiting for the device or gave up beginning the therapeutics. In order to investigate age differences, BMI, waiting time interval, PSG profile and sleep questionnaire features, the groups who began and did not begin the therapeutics were compared, verifying that there was no difference between such features (Table 3).

**Table 3:** Comparison of the participants who started the Continuous Positive Airway Pressure use with those who did not.

Aspect	Use of the CPAP		*p*
	No	Yes	
Age	61.7 ± 11.4	59.2 ± 12.6	0.552
BMI	34.8 ± 7.45	34.4 ± 6.8	0.741
Interval 01 (days)	211 ± 143	174 ± 90	0.234
Interval 02 (days)	134 ± 149	135 ± 156	0.956
Interval 03 (days)	222 ± 206	185 ± 156	0.392
Interval 04 (days)	90.4 ± 89.1	110 ± 155	0.481
AHI	40.5 ± 26.5	42.9 ± 26.1	0.571
OAI	14.8 ± 18.4	21.0 ± 22.3	0.183
ESS	13.2 ± 5.03	12.2 ± 5.0	0.209
PSQI	15.2 ± 7.58	17.5 ± 7.69	0.126

CPAP, Continuous Positive Airway Pressure; ESS, Epworth Sleep Scale; PSQI, Pittsburgh Sleep Quality Index; AHI, Apnea/hypopnea index; OAI, Obstructive Apnea Index; ODI, Oxygen desaturation index; Interval 01, Time between the polysomnography testing and the first medical reappointment; Interval 02, Time between the first medical reappointment and the CPAP titration; Interval 03, Time between the titration and CPAP prescription; Interval 04, Time between the CPAP prescription and the beginning of its use; Mann-Whitney statistical test, *p* < 0.05 (*).

## Discussion

In the current study, conducted at a reference public teaching hospital in the interior of São Paulo State, excessive waiting time was found for OSA diagnosis (T1) and CPAP prescription (T2), averaging 6.5- and 14.5-months respectively, a total of 21-months. National data like those, regarding mean waiting time for OSA diagnosis and treatment have been scarce in the scientific literature.

Long time intervals have also been observed in several studies from the most diverse regions in the world. One of them, conducted in Ontario, Canada, found mean time of 11.6 months (348 days) for the beginning of the CPAP treatment.^10^ Another study, conducted by Flemons et al. in 2004, assessed the time interval to undergo diagnostic PSG in five regions: United Kingdom, Belgium, Australia, United States and Canada. Countries with a public health system, such as Canada and United Kingdom, yielded waiting time ranging between 10- and 24-months, while in the United States, waiting time ranged from a few weeks at private clinics to 12-months at public or teaching institutions.^11^

Despite being excessively long, the time for diagnosing OSA found in this study follows the trend of other sleep services around the world. However, the calculation of this interval did not consider the time taken between the diagnostic suspicion in the primary healthcare level and the first appointment for its investigation at the specialized sleep service, which may have underestimated the value. Thus, waiting time may be longer than it was assessed.

One of the direct consequences of delaying diagnosis and treatment of that condition is the significant dropout rate observed along the diagnostic flow. Around 30% of the participants with referral for diagnostic OSA screening missed follow-up at some time during the process, and among them, over half of them (54.3%) did not undergo diagnostic PSG. In a study held in Canada in 2020, Thornton et al. found straight correlation between waiting time reduction for beginning the treatment and higher adherence to the CPAP, in addition to patients’ better clinical outcomes.^12^

Apart from the long, lengthy trajectory, other probable causes related to that high dropout rate in the service are the scarce information/valuing on the part of the population at large and the other medical specialties. Thus, certain lack of knowledge on the importance of its treatment is verified, as well as the difficulties raised by the high demand that the service has to manage, once it is the reference for 68 municipalities in the interior of São Paulo State, in addition to the limitation of the scheduling system.

However, to the participants who went all along the diagnostic flow, the waiting time did not warrant them the expected treatment. Only about 35% of those with CPAP referral could obtain the device after its prescription, while the remaining 65% are still waiting for the device or gave up beginning the therapeutics. Initially, the CPAP device would be granted by the UHS to all patients diagnosed with OSA. However, in practice, the device is not fully available due to its high cost. In order to obtain it, patients must request it to the Secretary of Health from their municipality, a process that entails time and bureaucracy, delaying and hindering the beginning of their treatment even more.

Some studies show that the low adherence to the treatment, as observed in this service, is more significant to low-income patients and users of the public healthcare system. This fact is associated with socioeconomic factors, low schooling and high cost of the device.^13^, ^14^ In the city of São Paulo, Zonato et al. identified greater rate of treatment after the diagnosis among patients being followed up in the private healthcare service when compared to those from the public healthcare service. It was also evidenced lower rate of treatment discontinuation in the former group.^15^

Despite the existing evidence on the adverse consequences of untreated OSA and the cost effectiveness in the use of the CPAP device in order to enhance clinical outcomes, public service users with OSA still face innumerable barriers along the process of diagnosis and treatment of this condition. It is necessary to implement measures that speed and facilitate the access to diagnosis, granting its proper treatment, thus reducing the multimortality related to that condition.

## Conclusion

The current study, conducted at a reference sleep service of a public hospital in the interior of São Paulo State, found long time interval in the diagnostic flow of OSA and difficulty in the access to the CPAP device in order to begin its treatment. These findings must encourage managers to invest in new patient-centered strategies that speed this flow and warrant patients the access to the CPAP to enhance the service efficiency in addressing that condition and reduction of the dropout rate along that process.

## Funding

Douglas Inomata Cardoso da Silva received a scientific initiation grant from the National Council for Scientific and Technological Development ‒ *Iniciação científica do Conselho Nacional de Desenvolvimento Científico e Tecnológico* (135235/2020-0) to carry out this project.

## Ethics statement

The study was approved by the local Research Ethics Committee (CAAE: 36451020.5.0000.5411).

## Conflicts of interest

The authors declare no have conflicts of interest.
